# Cone-Beam Computed Tomography Evaluation of the Mandibular Condyle and Articular Spaces Following Orthognathic Surgery Using Freehand Articulation Method in Patients With Class II and III Skeletal Deformity

**DOI:** 10.1155/ijod/4269097

**Published:** 2024-12-31

**Authors:** Amir Jalal Abbasi, Mojtaba Azadbakht, Farzaneh Mosavat, Mahsa Bayati

**Affiliations:** ^1^Department of Oral and Maxillofacial Surgery, Sina Hospital and Craniomaxillofacial Research Center, Shariati Hospital, Tehran University of Medical Sciences, Tehran, Iran; ^2^Department of Oral and Maxillofacial Surgery, Faculty of Dentistry, Tehran University of Medical Sciences, Tehran, Iran; ^3^Department of Oral and Maxillofacial Radiology, Faculty of Dentistry, Tehran University of Medical Sciences, Tehran, Iran

**Keywords:** articular space, cone beam CT, mandibular condyle, orthognathic surgery

## Abstract

**Objective:** This study aimed to assess the changes in the position and size of articular spaces and anteroposterior and mediolateral condyle dimensions following orthognathic surgery. Additionally, it evaluated the correlation between these changes and mandibular movement during surgery.

**Methods:** This experimental study examined 31 patients (16 with Class III and 15 with Class II malocclusions) who were candidates for orthognathic surgery. Bimaxillary orthognathic surgery was performed on 23 patients, while monomaxillary orthognathic surgery (mandible) was performed on 8 patients. Condyle positioning was achieved using the classic method. In pre- and postsurgical cone beam computed tomography (CBCT) scans, the anteroposterior and mediolateral dimensions of the condyle and spaces and the intercondylar angle were measured. The results were analyzed using the Wilcoxon signed-rank test, with a *p*-value of less than 0.05 considered significant.

**Results:** The medial and lateral condyle dimensions and the upper articular space did not change significantly after orthognathic surgery in both Class II and III groups. However, the posterior articular space dimensions showed a statistically significant reduction in both groups. Although the anterior articular space dimensions increased in both groups, this increase was significant only in the Class II group. Additionally, there was a significant relationship between the extent of mandibular advancement or setback and changes in both groups' anterior and posterior articular space dimensions and the upper articular space dimensions in the Class II group.

**Conclusion:** The classic method for condyle positioning is a suitable approach for orthognathic surgery. The most notable changes were observed in the anterior and posterior articular spaces, likely due to the backward force applied to the proximal part during the fixation stage. According to the evaluations and Spearman's rho, the likelihood of changes in anterior and posterior articular space dimensions increases with more significant advancement and setback.

## 1. Introduction

In orthognathic surgery, various methods have been suggested to correct maxillofacial discrepancies. The bilateral sagittal split osteotomy (BSSO) approach is the standard surgical technique for correcting jaw deformities [1]. The main advantages of the BSSO technique include ease of implementation, a low probability of unfavorable fractures and nerve damage, no need for skin incisions, and facilitation of the segment fixation process during surgery [1–3]. Over the years, several modifications of the BSSO technique have emerged, with significant changes introduced by Dal Pont. These modifications offer increased bone contact area between the proximal and distal parts, increased precision, and ease of repositioning the proximal portion [4]. In orthognathic surgery, optimal condyle positioning after surgical movement is crucial. Various studies have presented different methods to ensure correct performance [1–7]. *Condyle remodeling* is a continuous physiological process that provides permanent temporomandibular joint (TMJ) adaptation to forces during function. Significant alterations in condylar position may lead to malocclusion, and an early relapse risk due to the displacement of the distal segment once postoperative intermaxillary fixation (IMF) is removed [1]. Furthermore, an unfavorable position of the proximal segment and alterations in the condylar position inside the glenoid fossa after surgery can disturb the previously defined balance and create a new force distribution in the TMJ, negatively affecting long-term surgical stability [3, 8]. Alterations in the condylar position can also contribute to the development of temporomandibular disorders (TMD), including internal derangement and degenerative articular changes [3, 9, 10] and reduce chewing efficiency due to working and nonworking interferences [2, 3, 11].

*Condylar resorption* is defined as a progressive postoperative decrease in the condyle's shape, size, and total volume, which can be accompanied by a reduction in posterior facial height, retrognathism, and clockwise rotation of the condyle and ramus complex in severe cases [12]. The overall prevalence of condylar resorption has been estimated at 3%–15%, with a higher occurrence in the Class II group (severe mandibular hypoplasia) [8]. Influential surgical factors in condylar resorption include bimaxillary surgery, significant mandibular advancement, and high maxillary impaction [4, 8, 13]. Interestingly, no significant relationship has been found between the incidence of condylar resorption and preoperative symptoms of TMD or joint sounds over the presurgical period [8]. It is necessary to consider significant variations in TMJ morphology among individuals and notable differences in various subgroups of skeletal discrepancies [3, 4, 14]. In the Class III group, the condyle exhibits more anteroposterior elongation and anterior inclination than in the Class II group. Furthermore, in the Class III group, the glenoid fossa is broader and shallower, positioning the condyle closer to the roof of the glenoid fossa [3]. Regarding the condylar position in the glenoid fossa, the condyle is generally situated posteriorly in the Class III group. In contrast, the Class II group is positioned more anteriorly [3], which may influence both the mechanics of the TMJ and the risk of postoperative complications. The posterior positioning of the condyle in Class III patients may predispose them to different outcomes compared to Class II patients, necessitating tailored surgical approaches that consider these anatomical discrepancies. Furthermore, the differences in TMJ morphology underscore the importance of individualized monitoring and rehabilitation strategies postsurgery. Understanding these factors is critical for optimizing surgical planning and improving patient outcomes in orthognathic surgery.

Overall, the type of dentofacial deformity is correlated with changes in the condyle and glenoid fossa anatomy and the placement position of the condyle in the glenoid fossa. The type of dentofacial deformity can be explained by the differences in condyle loading patterns in various states of skeletal deformity [4, 14]. Despite the critical importance of condyle positioning during orthognathic surgery, several factors can reduce its accuracy, including the absence of a fixed definition of the centric relation [3, 4], removal of muscle tone under general anesthesia [11], and significant advancement in the hypoplastic mandible [3, 11]. Despite suggestions of various methods, including the condylar positioning device and the intraoperative awakening method, more consensus is needed on the preferred method to enhance the accuracy of condylar positioning.

This study aimed to assess the changes in the position and size of articular spaces and the anteroposterior and mediolateral condyle dimensions after orthognathic surgery and to evaluate the correlation between these changes and mandibular movement during surgery.

## 2. Methods and Material

In this prospective study, subjects consisted of 31 patients in the Class II and III groups who were candidates for orthognathic surgery and underwent a period of orthodontic treatment before surgery (Ethics code: IR.TUMS.DENTISTRY.REC.1398.120). Participants were included if they did not present with congenital syndromes or defects such as cleft lip and palate or velopharyngeal insufficiency (VPI), had no history of orthognathic surgery, and had completed a presurgical orthodontic treatment course. Exclusion criteria encompassed individuals with more than 3 mm maxillary impaction, more than 3 mm mandibular deviation, complex dentofacial deformities, and a history of previous trauma or fractures in the condyle and condylar neck area. Maxillary impaction and mandibular deviation were measured using standardized cephalometric radiographs with adjustments made for magnification analyzed by trained orthodontists. To ensure accurate measurements, a known-size calibration marker, such as a metal ball of a specific diameter, was placed on the patient's face near the image receptor, close to the midsagittal plane. The marker was positioned within the same plane as the anatomical landmarks being measured. Using imaging software, we calibrated the measurements by comparing the marker's size in the image with its actual known size, calculating the magnification factor, and adjusting the anatomical measurements accordingly. A maxillary impaction greater than 3 mm was determined by measuring the vertical distance from the incisal edge of the maxillary central incisors to the anterior nasal spine. Mandibular deviation exceeding 3 mm was assessed by measuring the horizontal displacement of the mandibular midline relative to the facial midline on frontal cephalometric radiographs. Complex dentofacial deformities were identified through comprehensive clinical examinations and reviews of the patient's medical and dental records. A history of previous trauma or fractures in the condyle and condylar neck area was ascertained through patient interviews and confirmed by reviewing medical records and imaging studies.

To determine the required sample size for our study, we used data from a pilot study that involved measuring the upper joint space dimensions in five patients from the Class III group. The preintervention mean (*μ*_pre) was 2.56, the postintervention mean (*µ*_post) was 2.77, and the standard deviation (SD) of the differences (SD_diff) was 0.27. Based on these measurements and using a Type I error (*α*) set at 0.05 and a study power (1−*β*) of 0.80, we calculated that 15 cases per group would be required to achieve statistically significant results.

The pilot study data provided a foundation for calculating the sample size based on clinically relevant differences observed in the dimensions of the upper joint space, ensuring that our sample size is adequate to detect meaningful changes.

Finally, 15 Class II and 16 Class III patients, aged between 19 and 34 years, were included in the study. Bimaxillary orthognathic surgery was performed on 23 patients, while monomaxillary surgery (mandible) was performed on 8 patients.

During orthognathic surgery, condyle positioning was achieved using the freehand articulation method. This method ensures the proper position of the condyle by applying a posterosuperior vector of pressure to the proximal part through a combination of hand pressure in the parasymphyseal area and pressure on the anterior border of the ramus using an anterior stripper, ensuring the absence of a step in the lower border; and maintaining tight contact between the proximal and distal parts. In both Class II and Class III skeletal discrepancy groups, the proximal and distal parts were fixed together using a 4-hole mini-plate with a profile of 1–1.4 mm. Postoperatively, patients underwent elastic therapy or a short period of IMF, depending on their conditions.

Cone beam computed tomography (CBCT) images were captured using the Acteon Whitefox CBCT scanner before surgery, and data were acquired using Planmeca Romexis Viewer with the standard protocol (80 KVP, 5 mA, 17 s of exposure, 0.3 mm voxel size, and FOV 170 × 200). The minimum period for repeating CBCT was 6 months after surgery, once the patient had returned to normal function and range of motion. CBCT was then performed again under the same conditions. Anterior, posterior, and mediolateral dimensions of the condyle, superior, anterior, posterior, medial, and lateral articular spaces, and the intercondylar angle were examined in both CBCT images by a radiologist. To ensure measurement reliability, intraobserver consistency was assessed using the intraclass correlation coefficient (ICC). The radiologist measured the superior, anterior, and posterior articular spaces in five Class II and five Class III patients before surgery, repeating the measurements within a 2-week interval. The resulting ICC of 0.83 indicated good reliability.

**Figure 1:**
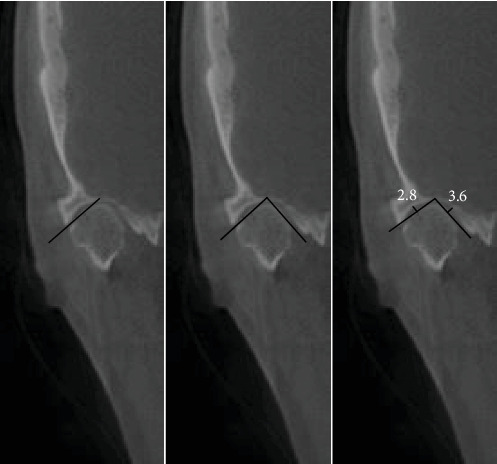
Assessment of medial and lateral articular spaces.

**Figure 2:**
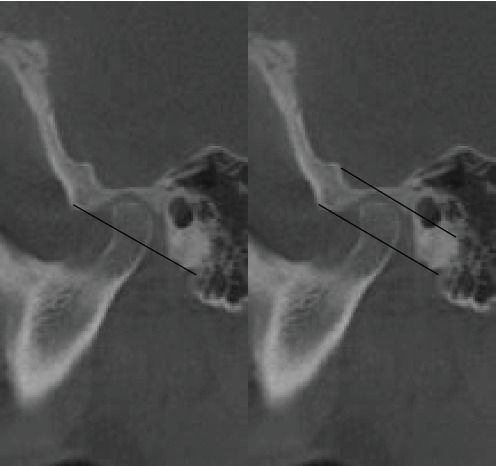
Drawing baselines to assess anterior, posterior, and superior articular spaces.

**Figure 3:**
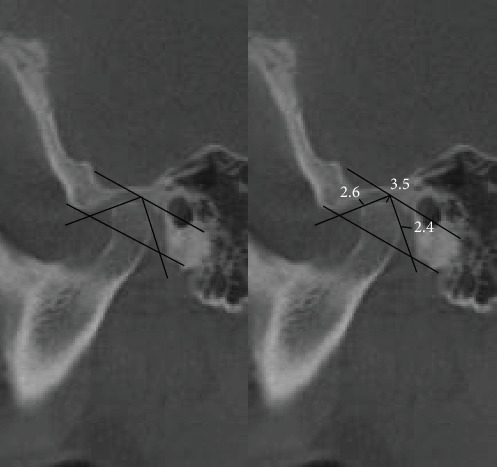
Completing the anterior, posterior, and superior articular space.

**Figure 4:**
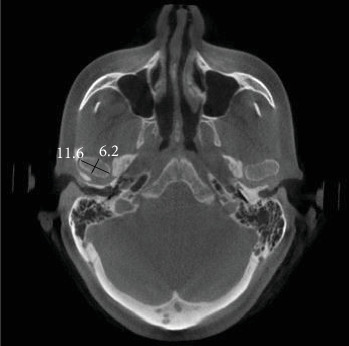
Measurement of anteroposterior and mediolateral dimensions of the condyle head.

**Figure 5:**
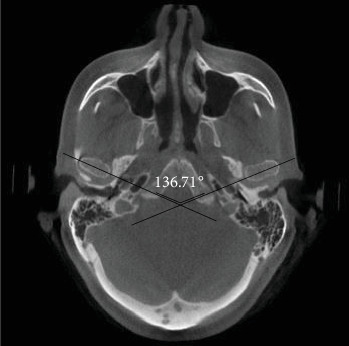
Evaluation of intercondylar angle in the axial cut.

In the first step of CBCT analysis, the panoramic axis was determined in the axial cut, designed to pass through the condyle's articular tubercle and the mediolateral axis. In the second step, to determine the dimensions of the medial and lateral articular spaces in the reconstructed coronal cut, two tangent lines were drawn from the highest point of the roof of the glenoid fossa to the most medial and lateral points of the condylar head borders. Perpendicular lines were then measured on the tangent lines at the medial and lateral borders of the condyle and the medial and lateral walls of the glenoid fossa (Figure 1). In the third step, CBCT sagittal cut analysis was used to measure the anterior, posterior, and superior joint spaces. First, a tangent line was drawn at the lowest point of the articular tubercle and the posterior projection of the eminence of the joint. Next, another line was drawn parallel to the previous line and tangent to the uppermost point of the glenoid fossa (Figure 2). Tangent lines were then drawn on the posterior and anterior borders of the condyle from the uppermost point of the glenoid fossa (the crossing point of the second line). Finally, the superior, anterior, and posterior joint dimensions were measured (Figure 3). In the fourth step, measurements of the axial cut of the scan were used to evaluate the anterior, posterior, and anteroposterior condyle dimensions (Figure 4). Also, the intercondylar angle was measured by drawing lines through the center of each condyle in axial cut, with the angle formed at a point near the midline of the mandible (Figure 5). Statistical analysis was conducted using SPSS software version 26. Due to the nonnormal distribution of the samples, the Wilcoxon signed-rank test, a nonparametric equivalent of the paired *t*-test, was used for paired comparisons in assessing the five articular space dimensions. Descriptive statistics were calculated to summarize the data, including means, SDs, and interquartile ranges (IQR). A *p*-value of less than 0.05 was considered statistically significant. In investigating the relationship between the degree of mandibular advancement or setback during orthognathic surgery and the changes in the study variables, the nonnormal distribution of the samples necessitated using a nonparametric method. Consequently, instead of employing Pearson's correlation, Spearman's rank correlation coefficient (Spearman's rho) was utilized to assess the correlation between the extent of mandibular advancement or setback and the study variables.

## 3. Results

The analysis revealed a slight, nonsignificant increase in the dimensions of the superior (*p*-value = 0.18) and lateral (*p*-value = 0.46) articular spaces in the postoperative period for Class II patients (Table 1). Similar results were reported for Class III patients regarding the superior and lateral articular spaces (*p*-value = 0.48 and *p*-value = 0.60, respectively) (Table 2). The dimensions of the anterior articular space increased in both Class II and III groups, with significance observed only in the Class II group (*p*-value = 0.007).

Additionally, a significant reduction in the posterior joint space was noted in both Class II (*p*-value = 0.009) and Class III (*p*-value = 0.013) groups. The medial articular space dimensions exhibited a slight, nonsignificant decrease in both Class II (*p*-value = 0.41) and Class III (*p*-value = 0.48) groups (Table 3).

Linear measurements of the mediolateral and anteroposterior dimensions of the condylar head demonstrated no significant changes in both groups (Tables 1 and 2). A minor, nonsignificant decrease in the intercondylar angle was observed in both Class II (*p*-value = 0.42) and Class III (*p*-value = 0.91) groups.

Overall, no significant differences were detected between the two skeletal discrepancy groups. The changes in condylar head dimensions (anteroposterior and mediolateral) and the intercondylar angle were minimal and nonsignificant in both Class II and III patients, with no significant difference between the groups (*p*-value = 0.57). Furthermore, no significant differences were observed between the two groups regarding the dimensions of the five joint spaces.

The analysis of the correlation between mandibular advancement or setback and articular space dimensions revealed a positive correlation between mandibular advancement in Class II (Spearman rho = 0.89) or setback in Class III (Spearman rho = 0.22) and changes in the dimensions of the upper joint space, with a more pronounced correlation in the Class II group (*p*-value = 0.0001).

For the medial joint space, Spearman rho was −0.18 in the Class II group and −0.04 in the Class III group. The negative Spearman coefficients indicate a decrease in the medial articular space in the preoperative and postoperative periods, with a more notable decrease in Class II patients. Although the dimensions of the joint space increased with advancement or setback amount in both groups, this relationship was insignificant (Table 4).

Regarding the lateral joint space, Spearman rho was 0.10 in Class II patients and 0.11 in Class III patients. The positive Spearman coefficients suggest a direct relationship between mandibular movement (advancement or setback) and the dimensions of the lateral joint space. However, this relationship was insignificant (*p*-value = 0.708 in Class II and *p*-value = 0.681 in Class III patients).

In evaluating the anterior joint space, Spearman rho was 0.85 in Class II patients and 0.73 in Class III patients. The positive Spearman coefficients indicate a direct relationship between the amount of advancement or setback and changes in the anterior joint space dimensions. This relationship was significant (*p*-value = 0.0001 in Class II and *p*-value = 0.0011 in Class III patients). For the posterior joint space, Spearman rho was −0.87 in Class II patients and −0.75 in Class III patients. The negative Spearman coefficients indicate a decreasing relationship between the amount of setback or advancement and the dimensions of the posterior joint space. This relationship was significant (*p*-value = 0.002 in Class II and *p*-value = 0.007 in Class III patients).

In correlation between mandibular movement and changes in the intercondylar angle, Spearman rho was −0.004 in Class II patients and −0.06 in Class III patients. The near-zero negative coefficients indicate no significant correlation between mandibular movement and changes in the intercondylar angle. The *p*-values (0.986 in Class II and 0.814 in Class III patients) further confirm the lack of a significant relationship between mandibular advancement or setback and changes in the intercondylar angle (Table 4).

## 4. Discussion

The lack of significant changes in the dimensions of the superior articular space in Class II (*p*-value = 0.18) and Class III (*p*-value = 0.48) patients after orthognathic surgery aligns with the findings of previous studies [11, 15]. Although some studies report varying results regarding changes in joint space dimensions, these variations are typically insignificant over long-term recall intervals (greater than 6 months) [6]. Despite applying a posterosuperior pressure vector to the proximal segment during the fixation stage, which could potentially reduce the dimensions of the superior joint space, the tonicity of the lateral pterygoid muscles and the anteromedial force exerted by these muscles prevent such a reduction. In the study by Lee et al. [11], a slight increase in the dimensions of the superior articular space was observed postoperatively; however, this increase was not statistically significant [15].

The current research results indicate a slight decrease in the dimensions of the medial articular space in both the Class II (*p*-value = 0.41) and III (*p*-value = 0.48) groups, which was statistically insignificant. These findings are consistent with previous studies [9, 12, 14], all of which reported statistically insignificant changes in medial articular space dimensions. Changes in condyle position within the horizontal plane (medial and lateral articular spaces) may result in transverse occlusal discrepancies, such as posterior cross-bite, accompanied by pain and discomfort during function.

Regarding the dimensions of the lateral articular space in this study, a slight, nonsignificant increase was observed in both Class II and III skeletal discrepancies (*p*-value = 0.46 and *p*-value = 0.60, respectively). Most studies also did not detect significant changes in the lateral joint space dimensions [12, 14, 16], except for the study by Sander et al. [9], which reported a 0.17 mm reduction in lateral articular space dimensions. Compared to the anterior and posterior articular spaces, the dimensions of the medial and lateral joint spaces were less affected by orthognathic surgery. This is attributed to the stabilizing role of the temporomandibular and collateral ligaments, as well as the lateral and medial pterygoid and masseter muscles, which help maintain the condylar head position in the mediolateral dimension.

The results also show an increase in the dimensions of the anterior articular space in both Class II and III groups, with statistical significance observed only in the Class II group (*p*-value = 0.007). Several studies have noted similar increases in anterior articular space dimensions [2, 4, 11, 15]. However, these increases tended to diminish and lose statistical significance at 6 months to 1 year postoperative, attributed to condyle remodeling and restoration of normal function and muscular tension.

The current study observed a significant decrease in the posterior joint space in both Class II (*p*-value = 0.009) and III (*p*-value = 0.013) groups, comparable to the alterations seen in the dimensions of the anterior articular space. Similar reductions in the dimensions of the posterior articular space have been reported in previous studies [2, 4, 6, 11]. Factors such as the patient's posture during surgery, gravitational effects, loss of masticatory muscle tonicity under general anesthesia, and the direction of pressure applied to the proximal part during fixation may contribute to the posterior positioning tendency of the condylar head, resulting in decreased dimensions of the posterior articular space and increased dimensions of the anterior articular space. Paralysis under general anesthesia has been noted to cause a 2 mm posterior repositioning of the condyle and a vertical drop in its position [11]. However, findings from Jung et al.'s study [6] suggest that changes observed in the dimensions of the posterior joint space in primary scans were reduced in secondary scans conducted at 6-month and 1-year intervals due to condylar remodeling. Returning to function and muscle tension may make these changes unstable over time. Most studies examining condylar position alterations postorthognathic surgery recognize the inevitability of such alterations and their impact on joint space dimensions [2, 9, 11, 13, 16]. However, the implications for TMDs and functional issues remain debated. Alternative methods, like certain condylar position guides, have not significantly improved skeletal stability or efficiency, often leading to longer surgeries and fixation stage challenges [1, 11].

The current study did not observe significant changes in linear measurements related to the mediolateral and anteroposterior dimensions of the condylar head, aligning with findings from Da Silva et al.'s study [4]. Additionally, no significant alterations were reported in condyle volume or condylar head dimensions postorthognathic surgery in this study. However, Jung et al. [6] noted that while there was a significant volumetric change in the posterior segments initially, this increase declined over time, with no significant difference from the original volume within 6–12 months postoperatively. This could be attributed to the posterior vector of force application during fixation, concentrating stress in this area. The presence of the lateral pterygoid muscle attachment in the anterior condylar head segment helps stabilize bone dimensions there.

Regarding changes in the intercondylar angle in both Class II and III groups, a slight and nonsignificant decrease was observed, consistent with findings from Vale et al.'s study [10]. Although Da Silva et al. [4] noted a relative decrease tendency in the intercondylar angle for the Class III group, the use of the submento-vertex modality for angle evaluation in this study may lead to less precise findings. Given the lack of change in medial and lateral joint space dimensions in the current study, the absence of significant intercondylar angle changes is expected, as temporomandibular and collateral ligaments prevent condylar head rotation in the glenoid fossa. Significant intercondylar angle changes are typically seen in severe cases of condyle degloving or poorly executed split types at the upper ramus level. The comparison of alterations between the Class II and III groups did not yield significance according to the obtained *p*-value (0.57). No significant difference was observed in the dimensions of the five joint spaces between these groups. In both groups, there were no significant changes in the upper, medial, and lateral joint space dimensions after orthognathic surgery, unlike the significantly reduced dimensions of the posterior articular space. Additionally, despite the increase in the dimensions of the anterior articular space in both Class II and III groups, this increase was statistically significant only in the Class II group. None of the reviewed studies assessed the correlation between the amount of mandibular movement and alterations in dimensions, and most did not compare Class II and III skeletal discrepancies. Moreover, none investigated the correlation between mandibular movement during orthognathic surgery and changes in joint space dimensions.

Due to nonnormal distribution, mandibular advancement or setback was analyzed using Spearman's Rho correlation coefficient. Postoperative alterations in the upper joint space dimensions were observed in both Class II and III groups but lacked clinical significance. A positive correlation between mandibular movement and changes in the upper joint space dimensions was notable, especially in the Class II group. However, the relationship between mandibular movement during surgery and changes in the upper joint space dimensions yielded different results. Spearman rho was 0.89 in Class II patients and 0.22 in Class III patients, indicating a positive relationship, with a significant *p*-value of 0.001 in Class II and 0.681 in Class III.

Regarding the medial joint space, both Class II and III groups showed postoperative reductions compared to preoperative, although not statistically significant. The negative Spearman coefficient indicates a reduction correlation, which is more pronounced in the Class II group. The lateral joint space increased slightly postsurgery in both groups but lacked statistical significance. Spearman rho for the correlation between mandibular movement and lateral joint space changes was 0.10 in Class II and 0.11 in Class III, showing a positive but insignificant relationship, with *p*-value of 0.708 and 0.681, respectively. However, a significant positive relationship existed between mandibular movement and changes in the anterior joint space dimensions.

Both Class II and III groups showed a significant decrease in posterior joint space dimensions postsurgery, correlating with mandibular setback or advancement. This might be attributed to upward and backward pressure on the proximal part and increased anterior joint space dimensions. Evaluation of the intercondylar angle in the Class II and III groups revealed a slight reduction postoperatively in both groups. However, this decrease did not reach statistical significance based on the *p*-value. The near-zero Spearman coefficient suggests a weak correlation, indicating no significant correlation between the amount of mandibular setback or advancement and intercondylar angle alterations.

Our study has unveiled several significant findings with promising clinical implications. The substantial reduction in the posterior joint space and the increase in the anterior joint space dimensions, particularly in Class II patients, indicate that mandibular advancement can bring about noticeable changes in the joint spaces. These findings, with their potential to enhance postoperative joint function and stability, are of great importance. Armed with this knowledge, surgeons can better plan orthognathic surgeries, leading to more favorable outcomes. Also, the positive correlation between mandibular advancement and changes in the superior joint space in Class II patients underscores the need for meticulous monitoring of joint space dimensions during and after surgery. The significant changes observed in the anterior joint space dimensions with mandibular advancement or setback underscore the potential to significantly improve patient comfort and function postoperatively.

Furthermore, the significant negative correlation between mandibular movement and posterior joint space dimensions underscores the importance of understanding how surgical adjustments affect joint spaces differently. This knowledge can inspire and motivate us to anticipate and manage potential postoperative complications related to joint space changes, leading to improved clinical practices. Our results suggest that while statistical significance is crucial, understanding the clinical implications of these changes is equally important. The findings of this study contribute to the body of knowledge on orthognathic surgery and provide valuable insights for improving clinical practices and patient outcomes.

Therefore, this study recommends conducting future research with a larger sample size and further specifying skeletal discrepancies beyond the Class II and III classifications. Expanding the sample size would enable more detailed subgroup analyses, enhancing the clinical relevance of the findings. Additionally, preparing a second postoperation scan at long-term intervals (after 1 or 2 years) is suggested to assess the compensation of changes recorded in the initial postoperation scan and the impact of continued function after surgery on condyle position for more precise results.

## 5. Conclusion

The results highlight significant changes in the anterior and posterior articular spaces, likely attributed to the backward force applied to the proximal part during the fixation stage. Based on the evaluations and Spearman rho analysis, the likelihood of alterations in the dimensions of the anterior and posterior articular spaces increases with higher mandible advancement or setback. This study also examined the relationship between mandibular movement during orthognathic surgery and found that as the mandible's advancement or setback increased, so did the probability of changes in the dimensions of the joint spaces, especially in the anterior, posterior, and upper articular spaces. Despite observing mainly minor alterations in the dimensions of the five joint spaces in this study, with significant changes in the anterior and posterior articular spaces, none of the patients experienced immediate relapse, functional issues, or temporomandibular pathologies.

## Figures and Tables

**Table 1:** The result of measuring variables in class II before and after surgery.

Variables		Range (min–max)	Mean (standard deviation)	*p*-Value
Upper articular space	Pre-Op	2.3–2.6 mm	2.46 mm (0.089)	0.18
	Post-Op	2.3–2.7 mm	2.46 mm (0.089)	

Anterior articular space	Pre-Op	1.9–2.3 mm	2.10 mm (0.103)	0.007
	Post-Op	2–2.7 mm	2.31 mm (0.192)	

Posterior articular space	Pre-Op	2.8–3.3 mm	3.04 mm (0.140)	0.009
	Post-Op	2.7–3.3 mm	2.94 mm (0.176)	

Medial articular space	Pre-Op	2.5–3.3 mm	2.87 mm (0.186)	0.41
	Post-Op	2.6–3.1 mm	2.84 (0.168)	

Lateral articular space	Pre-Op	3.4–3.9 mm	3.63 mm (0.161)	0.46
	Post-Op	3.4–3.8 mm	3.62 mm (0.109)	

Intercondylar angle	Pre-Op	117°–139°	132.5° (3.997)	0.42
	Post-Op	115°–135°	131.5° (3.778)	

Anterior–posterior dimension of condyle	Pre-Op	4.8–6.1 mm	5.42 mm (0.338)	0.73
	Post-Op	4.5–6.2 mm	5.42 mm (0.444)	

Mediolateral dimension of condyle	Pre-Op	10.4–12 mm	11.35 mm (0.502)	0.68
	Post-Op	10.4–11.9 mm	11.32 mm (0.479)	

**Table 2:** The result of measuring variables in class III before and after surgery.

Variables		Range (min–max)	Mean (Standard deviation)	*p*-Value
Upper articular space	Pre-Op	2.3–3.1 mm	2.52 mm (0.179)	0.48
	Post-Op	2.3–3 mm	2.52 mm (0.163)	

Anterior articular space	Pre-Op	1.7–2.3 mm	2.01 mm (0.160)	0.20
	Post-Op	1.7–2.7 mm	2.13 mm (0.237)	

Posterior articular space	Pre-Op	2.7–3.3 mm	3.00 mm (0.147)	0.013
	Post-Op	2.5–3.3 mm	2.89 mm (0.200)	

Medial articular space	Pre-Op	2.4–3.1 mm	2.81 mm (0.199)	0.48
	Post-Op	2.4–3.1 mm	2.87 mm (0.194)	

Lateral articular space	Pre-Op	2.9–3.9 mm	3.40 mm (0.307)	0.60
	Post-Op	2.7–3.8 mm	3.41 mm (0.307)	

Intercondylar angle	Pre-Op	117°–135°	125.3° (4.855)	0.91
	Post-Op	113°–134°	124.5° (4.356)	

Anterior–posterior dimension of the condyle	Pre-Op	4.7–6.4 mm	5.69 mm (0.419)	0.56
	Post-Op	4.4–6.5 mm	5.67 mm (0.483)	

Mediolateral dimension of the condyle	Pre-Op	10.4–12.2 mm	11.20 mm (0.470)	0.87
	Post-Op	10.4–12.1 mm	11.19 mm (0.403)	

**Table 3:** *p*-Values comparing the articular space dimensions.

Comparison before and after surgery	*p*-Value	Second quartile (P50) Before/after surgery	Third quartile (P75) Before/after surgery
Upper articular space in Class II	0.18	2.5/2.5	2.6/2.5
Upper articular space in Class III	0.48	2.55/2.55	2.7/2.7
Anterior articular space in Class II	0.007	2.3/2.1	2.4/2.2
Anterior articular space in Class III	0.2	2/1.9	2.1/2.05
Posterior articular space in Class II	0.009	2.8/2.9	2.9/3
Posterior articular space in Class III	0.013	2.9/3	3/3.1
Medial articular space in Class II	0.41	2.8/2.9	3/3
Medial articular space in Class III	0.48	2.7/2.8	2.8/2.9
Lateral articular space in Class II	0.46	3.6/3.6	3.7/3.7
Lateral articular space in Class III	0.60	3.45/3.45	3.45/3.45

**Table 4:** Correlation of changes in articular space dimensions with mandibular movement.

Type of correlation	Spearman rho	*p*-Value
Changes in anterior articular space relative to mandibular setback	0.73	0.0011
Changes in anterior articular space relative to mandibular advancement	0.85	0.0001
Changes in posterior articular space relative to mandibular setback	−0.75	0.007
Changes in posterior articular space relative to mandibular advancement	−0.87	0.002
Changes in upper articular space relative to mandibular setback	0.22	0.405
Changes in upper articular space relative to mandibular advancement	0.89	0.0001
Changes in lateral articular space relative to mandibular setback	0.1	0.681
Changes in lateral articular space relative to mandibular advancement	0.10	0.708
Changes in medial articular space relative to mandibular setback	−0.04	0.868
Changes in medial articular space relative to mandibular advancement	−0.18	0.499

## Data Availability

The data are available from the corresponding authors upon reasonable request.
